# Exploring the vulnerability of practice-like activities: An ethnographic perspective

**DOI:** 10.3389/fsoc.2022.1003741

**Published:** 2022-12-14

**Authors:** Yemisi Bolade-Ogunfodun, Matthew Sinnicks, Kleio Akrivou, Germán Scalzo

**Affiliations:** ^1^Henley Business School, University of Reading, Reading, United Kingdom; ^2^Southampton Business School, University of Southampton, Southampton, United Kingdom; ^3^Business Ethics Department, School of Economics and Business, Universidad Panamericana, Mexico, Mexico

**Keywords:** practices, management, ethnography, community, friendship, MacIntyre, internal goods, workplace community

## Abstract

**Introduction:**

This paper explores the vulnerability of practice-like activities to institutional domination.

**Methods:**

This paper offers an ethnographic case study of a UK-based engineering company in the aftermath of its acquisition, focusing in particular on its R&D unit.

**Results:**

The Lab struggled to maintain its practice-based work in an institutional environment that emphasized the pursuit of external goods.

**Discussion:**

We use this case to develop two arguments. Firstly, we illustrate the concept of “practice-like” activities and explore their vulnerability to institutional domination. Secondly, in light of the style of management on display after the takeover, we offer further support to MacIntyre's critique of management. Finally, based on the empirical data we reflect on the importance of organizational culture, as well as friendship and the achievement of a common good in business organizations for these kinds of activities.

## Introduction

This paper offers an ethnographic case study of a UK-based engineering company in the period after it was acquired by a large American multinational company, focusing in particular on its R&D unit, known as “the Lab,” and the wider organization. The analysis of the data revealed the Lab's struggles to maintain its practices and internal goods in an institutional environment that emphasized the pursuit of external goods. We draw on this case to develop two central arguments.

Firstly, this paper aims to illustrate the tension that often arises between practices and institutions, and in particular the vulnerability of “practice-like” activities to institutional domination. The concept of “practice-like” activities has been appealed to in previous work on MacIntyre and organizations (Moore and Beadle, 2006; Beadle, 2008), and in this paper we explore this notion in detail. The operation of the Lab was certainly practice-like, yet it lacked the additional features of fully-fledged or “paradigmatic” practices (Sinnicks, 2019), features which render such practices less vulnerable to institutional domination.

Secondly, in light of the concerns of management after the takeover, this paper aims to add further support to MacIntyre's (2007) critique of management as amoral. While this has been taken up in theoretical contributions to the literature (e.g., Beadle, 2002; Knight, 2017), applications of MacIntyre's work have typically sought to identify and explore examples of work where manipulative and emotivistic management is absent, or at least mitigated by commitment to goods internal to practices (Moore, 2012a; Beadle, 2013). However, the organization at the heart of this study provides an illustration of the consequences of an indifference to, and at times an apparent unawareness of, internal goods.

Based on the empirical data, the paper also reflects on how important the organizational culture and the virtue of friendship are, linked with the achievement of a common good in business organizations for these kinds of activities and their relevance in research in business ethics inspired by MacIntyre's (1999, 2004), within which friendship is a key theme. In this study, friendship is undermined by the kinds of changes experienced by the Lab. Friendship in the workplace is a key facet of flourishing and yet something that is, itself, vulnerable to the contingencies of contemporary working life. In addition to bad management, these include abrupt shifts in working practices (Sennett, 1998), inequalities of power (Sinnicks, 2020), and so on. The paper also argues that in addition to the emergence of scholarship that explores aspects of MacIntyre's thought other than his conception of practices (Such as Bernacchio, 2018; Couch and Bernacchio, 2020; Burton and Sinnicks, 2022; Pinto-Garay et al., 2022; Wightman et al., 2022), it is worth focusing more closely on management outside of practices. This stems from a recognition that non-practice-based workplaces exist, and still need good management, an observation that informs MacIntyre's (2016) most recent comments on work. Indeed, “practice-like” work is often closer to non-practice-based work than it is to paradigmatic practices, which suggests that appeals to distinctive internal goods, and a framing of managers as protectors of practice, are not universally applicable. Finally, it also suggests a closer engagement with other methodologies and areas of concern in the study of work and organizations, in this case ethnography, as such an approach is complementary to the existing vein of empirical research on MacIntyre, business ethics, and organizations (see, for example, Moore, 2012b; Dawson, 2015; Robson and Beadle, 2019; Chu and Moore, 2020; Nicholson et al., 2020).

## Practices, institutions, and management

Alasdair MacIntyre has had a remarkable impact within business and organizational ethics (Ferrero and Sison, 2014; Akgün et al., 2022). In this section we critically review the concepts of practices, institutions, and management in MacIntyrean research, which have been central to this impact, with a particular focus on the nature of practice-like work.

MacIntyre's provides the following definition of a practice:

a coherent and complex form of socially established co-operative human activity through which goods internal to that form of activity are realized in the course of trying to achieve those standards of excellence which are appropriate to, and partially definitive of, that form of activity, with the result that human powers to achieve excellence, and human conceptions of the ends and goods involved, are systematically extended. (2007, p. 187)

MacIntyre also provides a number of examples to illustrate the concept (2007, p. 187). Architecture, chess, portrait painting, physics, football, and farming are practices and are therefore both in possession of their own distinctive goods, and conducive to virtue acquisition and its exercise. On the other hand, bricklaying, throwing or kicking a ball with skill, and planting turnips are examples of non-practices, and so lack the goods and connection to moral education. Because of the nature of internal goods, only those with experience of the practice in question can properly be said to understand its goods (MacIntyre, 2007, p. 189).

The contrast to the concept of internal goods, which are only available *via* engagement in some practice or other, is external goods, which can always be obtained in a variety of ways, and include such things as money and power. Because of this focus, institutions have a tendency to undermine practices. Their focus on external goods means institutions are liable to an acquisitiveness which can have a corrupting effect on practices (Moore and Beadle, 2006), particularly under the conditions of Anglo-American style capitalism (Keat, 2008).

Nevertheless, it is important to remember that practices need institutions. As MacIntyre puts it, institutions

are involved in acquiring money and other material goods; they are structured in terms of power and status, and they distribute money, power and status as rewards. Nor could they do otherwise if they are to sustain not only themselves, but also the practices of which they are the bearers. For no practices can survive for any length of time unsustained by institutions. (MacIntyre, 2007, p. 194)

However, the institutional requirement does not imply that institutions are needed in the same way, or to the same degree, by all practices. Of course, institutions are needed to safeguard practices if they are to fully flourish, but paradigmatic practices, i.e., those most obvious examples of practices which hardly admit room for disagreement, are often clearly available outside of institutions, and indeed often attract devoted amateurs capable of engaging in the activity to a high level. Examples such as the hermetic philosopher, or the excellent chess player who has never joined a club, suggest practices can—could—survive and do well with very little institutional support, even if not perhaps flourish as clearly as they would with excellent institutions. The clubs, tournaments, and ranking bodies are required for the highest achievement in chess, and thus for the practice as a whole to develop historically, but nevertheless the isolated pair of chess playing friends might still engage quite seriously even if, in the wake of a chess-related disaster, say, its institutions disbanded. Another example, the “exploration of wilderness” (MacIntyre, 2007, p. 275) is clearly distinguishable from any particular set of institutional frameworks.

There are, of course, practices that are heavily dependent on institutions such as medicine and other professions, which require institutions related to training, accreditation, provision of services and so on, as well as practices that are even conceptually indistinguishable from a kind of institution, for instance, the making and sustaining of family life (MacIntyre, 2007, p. 188). But it is worth noting that there is a good degree of variation, and that sometimes the required acquisition of external goods in order for a practice to survive is relatively meager. Let us not forget that, while practices typically need institutions to survive and almost always need institutions in order to flourish, practices themselves inspire us to establish practice-sustaining institutions. For all that poetry owes to poetry-sustaining institutions, those institutions owe yet more to poetry.

Practices play a central part in human flourishing. This is in part because of the goods internal to practices, and in part because of how these internal goods relate to our moral development: it is only through acquiring and exercising the virtues that we can properly experience the internal goods practices make available. Certain virtues play a role in all, or almost all practices. Examples include justice, truthfulness, constancy, and courage. As a result, engagement in practices plays an important role in character development: the person engaged in a practice “is perfected through and in her or his activity” (MacIntyre, 1994, p. 284).

Practices are ubiquitous features of human life even in societies in which they occupy a relatively marginalized position. The ubiquity of practices allows the traditions of the virtues to survive in the lives of ordinary people. This is because, for instance, only through being prepared to give and receive honest criticism, and cultivate self-honesty, can we come to experience the satisfactions practices can provide. As MacIntyre says, “the exercise of the virtues is something learned in the context of practices… those who engage in practices need the virtues if they are to achieve the individual and common goods internal to practices” (MacIntyre, 2013, p. 216). As a result, practices are intrinsically satisfying, inherently worthwhile, and also morally educative. In this way, just as virtues are internal rather than external means to the end of human flourishing, practices are internal means to acquire and exercise virtue. Most applications of the concept in the business ethics literature have focused on arguing for the inclusion of some activity as a practice. Inevitably, the focus has been on marginal cases. Very obvious examples of practices such as painting, poetry, and philosophy need no such defense, whereas activities such as investment advising (Wyma, 2015), accountancy (Francis, 1990; West, 2018), and finance (Sison et al., 2019; Roncella and Ferrero, 2020; Rocchi et al., 2021) may seem, on the face of it, to lack the richness of more paradigmatic examples, or to have an ambiguous status. As a result, arguments in favor of understanding such activities as practices are more likely to possess the novelty and contentiousness that are valued in academic research.

However, we might worry about this leading to an excessively permissive understanding of practices. Showing that surprising cases do, in fact, answer to MacIntyre's definition of a practice is valuable, but that value would be diminished if it were almost impossible to exclude any activity, no matter how humdrum or contentious. One-way of dealing with this potential problem, is to invoke the concept of “practice-like” activities (appealed to by, for instance, Beadle and Knight, 2012; Moore, 2017), a move which also has the advantage of allowing for applications of MacIntyre's “virtues-goods-practice-institution” schema (Moore and Beadle, 2006) which do not require every scholarly contribution to engage in a detailed defense of some activity or another as a practice.

Thus, it is worth paying attention to the concept of “practice-like,” which refers to activities which possess some but not all of the characteristics MacIntyre outlines in his definition of practices, or which possess the characteristics in so partial or qualified a way that might discourage us from accounting them full practices. This worthiness of attention stems in part from the fact that the concept of the “practice like” is intrinsically puzzling. Almost all activities are practice-like in some way, and so it is impossible to specify exact limits. MacIntyre's examples of non-practices—turnip-planting, bricklaying, and throwing a ball with skill (2007, p. 187)—are all sufficiently practice-like to have corresponding practices: farming, architecture, and football (2007, p. 187). We recognize that turnip-planting is like farming, which is why the sets of examples possess a pleasing symmetry.

However, even very dissimilar things are alike to at least some degree, and so it is worth paying attention to the ways in which so-called practice-like activities do or do not possess the distinctive internal goods, and attendant intrinsic satisfactions and moral education, associated with practices. Thus, the boundaries of the concept of practices remain a worthy object of reflection and research, even if these boundaries will inevitably remain somewhat blurry (Sinnicks, 2019). This blurriness is particularly inevitable for the concept of the “practice-like,” because things can be like practices in different ways. If someone were to invent a new game one afternoon, the game might possess rich internal goods, but it would lack the social establishment and communal aspects of fully-fledged practices. On the other hand, turnip planting is socially established, and can give rise to a supporting community, it just lacks the rich internal goods. Thus, applications of the concept of “practice-like” may vary, just as the concept of “maze-like” may equally apply to buildings, dense parts of forest, or works of visual art, albeit in very different ways.

However, it remains worth paying attention to the concept of practice-like activities themselves, both because they are very widespread and because, as we discuss below, they seem particularly vulnerable to becoming subordinated to institutions as a result of lacking the obvious appeal of fully-fledged practices. This status is liable to make them less often sought out by would-be practitioners, and their goods less visible to the uninitiated.

In order to explore the concept of practice-like activities further, let us start with an activity that falls on the “wrong side” of the practices/non-practices divide: business, which has been analyzed as a putative practice by several commentators (e.g., Moore, 2002; Dawson and Bartholomew, 2003). In response, Beadle (2008) has offered a compelling case against conceiving of business as a practice (see also MacIntyre, 2008). However, there is one aspect of Beadle's argument which we take to be instructively mistaken. Beadle claims that one of the reasons that business cannot be a practice is that the “coherence of MacIntyre's definition of practice would be undermined if the same set of practitioners can coherently simultaneously engage in two practices. They cannot” (Beadle, 2008, p. 238).

However, we can readily imagine a parent getting home from a long trip and immediately playing chess with a child in order to give the tired and frazzled other parent some respite. In so doing they are participating in both the practice of chess and in the making and sustaining of family life, both examples of MacIntyrean practices. Such practices remain conceptually distinct *qua* practices, and yet they can be engaged in simultaneously. Indeed, MacIntyre notes the possibility of actions falling under different and yet equally accurate descriptions and gives the examples of someone “‘Digging,' ‘Gardening,' ‘Taking exercise,' ‘Preparing for Winter' or ‘Pleasing his wife”' (2007, p. 206). In the example we have provided of engaging in the practices of chess and family life, it seems entirely possible that someone can simultaneously, and correctly, understand him or herself as participating in both practices.

The observation that multiple practices can be engaged in simultaneously opens up the possibility of engagement in practice-like activities as well as having some other more central objective. In such cases, the practice-like activity might never quite attain the centrality to someone's life that serious engagement in fully-fledged practices is typically thought to demand because it is secondary to some other concern, such as the enjoyment of the sociable aspects of the workplace, or to sustaining a workplace community.

Yet, even where secondary to this focus on community and friendship, the practice-like activity might retain an important place in an agent's motivational set. After all, most forms of work seem to fall short of painting, physics or poetry and yet are clearly not ethically inert. Such activities can importantly shape workplace relationships, and the intrinsic enjoyment of work, without us having to attempt to analyse their goods with the kind of focus warranted by paradigmatic practices. However, in such cases, we can still recognize that the contours and nuances of the activity play an important role.

This understanding of practice-like work allows us to further recognize that participating in some form of practice-like work (A) with commitment to excellence in that form of work is often most centrally a way of participating in the relevant community, so that if the organization that houses A changed direction to emphasize a new, perhaps related, product or service such that employees became engaged in a different practice-like activity (B), A would be replaced with B, but the community would remain unchanged, and a variety of largely generic virtues—diligence, honesty, fairness, soundness of judgement, collaborativeness, and practical wisdom—would need to be exhibited in the engagement with B in order to preserve the peer-recognition and workplace relationships required for the enjoyment of the community. Thus, the community is central, and yet it presupposes a serious engagement in either A or B equally. Without some practice-like form of work to structure the working relationships, the community would be entirely unavailable.

The practice-like form of work, A or B or whatever, is engaged with in a way that partially reflects a commitment to their practice-like good—and note that such a story may be unavailable where the work is the most obviously un-practice-like drudgery—but may instead be principally motivated by the pursuit of the goods of community membership. So the goods of the work are largely external insofar as they are generic goods available to an extremely wide range of occupations, such as “purpose, belongingness, and identity” (Michaelson et al., 2014, p. 77), and yet it is the particularities of that particular workplace, of activity B, say, that allows us to really understand how internal goods, goods that cannot be achieved in any other way, enter into the picture. In this way, the community is playing an especially central role in the ethically salient aspect of the work (see Sinnicks, 2021).

However, it is important to note that practice-like activity A can be effectively substituted by B only if the transition between them is achieved in a way that does not undermine the central community. However, it is very easy for those managers driving the shift from A to B to be entirely insensitive to, perhaps even largely unconscious of, the goods of the community, such that undesirable changes to the friendship bonds in that community are an unintended consequence of the shift in “core business.”

This insensitivity to goods is implied by MacIntyre's critique of management as amoral. On this front, MacIntyre argues that

the manager represents in his character the obliteration of the distinction between manipulative and non-manipulative social relations… The manager treats ends as given, as outside his scope; his concern is with technique, with effectiveness in transforming raw materials into final products, unskilled labor into skilled labor, investment into profits. (2007, p. 30)

As a result, the manager is incapable of genuine moral engagement, and instead embodies emotivism, the “boo-hurrah” theory of moral meaning associated with Ayer (1936) and Stevenson (1944). According to this theory, moral claims are merely expressions of subjective feeling. Thus, to say “X is wrong” is simply to say, “I dislike X, do so as well.” The result is an obliteration of the distinction between manipulative and non-manipulative social relations. This is because any way of bringing someone to disapprove of X is equally legitimate: rational persuasion has no privileged position over rhetorical trickery because there is no way to rationally appraise such claims. Accordingly, any arising manipulativeness is not an ethical failure, but rather an unavoidable feature of the managerial role.

While Moore suggests that it “seems clear that the basic tenets of [MacIntyre's] position, at least in respect of managers in business organizations under Anglo-American capitalism, remain in place” (Moore, 2008, p. 495), he regards MacIntyre's broader critique of management as being mistaken: “Are managers simply the morally-neutral efficient achievers of predetermined ends? Clearly... the answer is no” (2008, p. 505). In workplaces that house a fully-fledged practice, and when in good order, management is concerned to institutionally support the practice. In such a situation, MacIntyre acknowledges that “[m]anagers become enablers” (2016, p. 132), and as a result of both this, and of MacIntyre's broader account of institutions, management has been understood as a practice (Brewer, 1997) or a “domain-relative” practice (Beabout, 2012).

However, in more marginal cases, where the work is practice-like, and thus where it may not attain the centrality of one's life often associated with engagement in fully-fledged practices, the community aspect may become more important than the nature of the work itself. Under such circumstances, supporting the practice-like activity may become less central to the manager's task; and facilitation of community *qua* community can become more central precisely because that community has become more central to the workers, and a more powerful determinant of their commitment and motivation. The facilitation of community is itself a practice, that of politics, in its Aristotelian rather than its modern sense, according to MacIntyre (2007, p. 195), see also Sinnicks (2014). Given that MacIntyre has placed a great emphasis on the importance of local, small-scale communities (1999, p. 142), the face-to-face interactions that take place within them, the relationships that are being developed, clearly this community aspect is central to MacIntyrean understandings of the contemporary workplace. While MacIntyre has been heavily critical of corporate modernity (MacIntyre, 1979; see also McMylor, 1994), some commentators have been keen to stress that communities can be virtual rather than face-to-face and that the modern firm is capable of housing communities (Dobson, 2008), and even that such modern communities have a variety of advantages over pre-modern communities (Dobson, 2009). Whether contemporary workplaces face particular challenges when it comes to achieving the status of MacIntyrean community, that they facilitate participation (Bernacchio, 2021) is a decisive factor. This is in part because of another facet of MacIntyre's critique of management: his rejection of a distinctively “managerial expertise”. In commenting on claims to such expertise, MacIntyre says, “those who arrogate to themselves an exclusive, professionalized authority of a certain kind by that very act of arrogation discredit their own claims to legitimate authority” (2006, p. 51). Such claims are entirely incompatible with the kinds of participative communities MacIntyre regards as being a precondition of human flourishing.

Having reviewed the central features of MacIntyre's thought that pertain to flourishing in the workplace, we next turn to the task of documenting the vulnerability of practice-like work in our empirical case study of a tech firm takeover. We use the example of the Lab to explore our understanding of practice-like work, and to illustrate how it is subject to external pressures through the pertinent example of a takeover as organizational change. The next section details how the research was carried out.

## Methodology and organizational context

To explore our understanding of practice-like work, we studied a post-acquisition context where a small hi-tech firm (pseudonym is Brownfield) was acquired by a large multinational (pseudonym is Alpha-D) in 2012. The focal context for the study comprises the Research and Development (R&D) engineering unit known as “the Lab.” The study of the acquisition covered a six-month period of continuous presence and embeddedness in the field in 2015, including a two-week break incorporated halfway into the study to allow for some critical distance and reflexivity for the researcher. Overall contact was however maintained with the field site over a two-year period between 2015 and 2017. The six-month period of continuous study coincided with the integration phase in the organization. An ethnographic approach was adopted for the study and this provided an emic view of the day-to-day operations of the Lab. Ethnography as a research method allows for developing familiarity with the context such that one is able to observe interactions between different organizational cultures and systems of meaning. Specifically, the acquisition was two-and -a-half years into the integration phase and this time period provided opportunity to examine interactions between the two entities and the processes of adjusting to the organizational change. This is particularly relevant for the Lab as its core work entails high levels of interdependence, creativity as well as autonomy in producing and maintaining a range of products based on its specialist technology. Access was secured through the heads of HR and R&D in the acquiring company and information sheets were given, with consent forms signed at the organizational level but also at the level of the Lab to explain the research and provide assurance of confidentiality and anonymity. A non-disclosure agreement issued by the acquiring company was also signed by the ethnographer.

As is typical with ethnographic studies, data was generated through several sources including participant observation, in-depth interviews and analysis of company textual records and documents. Data on observation were documented in field notes and recorded as close to the times of occurrence as possible. The field notes were then used to develop ethnographic narratives of the context. Apart from general observations of day-to-day patterns in the context, including informal gatherings, lunchtime routines and special or one-off events, a total of 56 meetings were observed as Lab team leaders met weekly and with other departments over the six-month period of continuous observation. Most of the meetings ranged between 1 and 2 h in length.

Data from interviews were recorded where permitted by participants and later transcribed. Interviews were conducted with 18 volunteer participants from the six subunits which made up the Lab and across different job functions to give a representative perspective. The interviews ranged from 50 to 90 min per participant and gave the opportunity to probe the values that were integral to the nature of the Lab's engineering work and its historical work patterns. A range of organizational documents were examined to elicit a historical perspective of the acquirer's culture and work values. These included historical records of the acquirer's history over several decades of its existence, articles featured in both print and electronic media as well as press reports. The integration of participant observation, interviews and analysis of organizational documents enabled us to build a picture of the practices and work culture in the post-acquisition context of the Lab. In particular, areas of cultural incongruence were made prominent as the engineering team culture interacted with the newly imposed bureaucratic structure of the acquirer. The findings that follow highlight the contrast between the practices in the Lab and its role in the experience of its members, and the larger organization.

Prior to acquisition by Alpha-D, the Lab was one of five specialist businesses which had been acquired by Brownfield—a US-headquartered multinational company with a UK subsidiary. The acquisition of the five companies gave Brownfield expertise covering complementary technologies and applications. The Lab then became known as the engineering team in Brownfield and was renowned for its expertise in its industry, developing hi-tech products useful in the transportation sector and for access control.

In Brownfield, the engineering team carried on their operations as before with no visible change to their relatively flat organizational structure until 2012. Traditionally, their work was divided by subject areas although they collaborated to produce functioning products. The team developed the physical equipment and the software which ran on it. They were supported by colleagues who tested the equipment for performance, customer service staff who took customer orders and handled administration and a technical support team who integrated product components at the customer's site, set them up to run and serviced the products regularly as specified in the sale contract agreement. The team had been managed by a technical manager and an engineering manager who reported to the managing director.

Historically, the engineering team had worked interdependently, relatively informally and decision-making was relatively quick, given the few layers of management. Data from interview participants revealed their recollection of the speed of access to information, approvals, funding and equipment to facilitate their work prior to and after the acquisition by Brownfield. Participants also expressed during interviews that the relatively small number of employees had historically facilitated closeness, communication, and collegial personal interaction due to a lack of bureaucratic elements in their organizational structure.

Alpha-D (the acquirer) is a US-based multinational company which has been in existence for many decades. Since its inception, it has grown in size and in business scope through international expansion *via* independent subsidiaries in different continents. Historically, Alpha-D specialized in the manufacture and marketing of a wide range of materials for industrial, commercial, and personal use. The company was highly structured and organized along core activities such as research and development, manufacturing, marketing and sales, warehousing, shipping, administrative, and other support services. R&D—through which new products and possibly entirely new industries could be developed—is at the core of product innovation in the company.

Alpha-D acquired Brownfield in 2012 and as expressed by the Lab leader and other Lab members during interviews, they indicated that the acquisition transaction was effected without the involvement of the engineering team. They had felt disconnected from the process because the owner of Brownfield had held strategic acquisition talks with Alpha-D and decision-making did not trickle down to the team level. Following acquisition, the business was then grafted into the UK subsidiary of Alpha-D.

Alpha-D's focus on continuous pursuit of R&D was driven by expectations of profitability and strengthening its competitive position. Examination of Alpha-D's published organizational biography indicates that the company's first profitable product emerged just over a decade after inception but was threatened by a legal challenge by a competitor over patent infringement. This placed the company in a vulnerable position, which put profitability, product ownership, and company reputation at risk. This significant experience in the early years of the parent company's history contributed to the company's sensitivity to profitability, reputation, and intellectual property. Processes were thereafter instituted to account for resources channeled to development work (to promote efficient usage) and to protect every technology or idea. Mirroring the parent company, Alpha-D UK continued with structures, policies and procedures to track product profitability as returns on R&D spending. This was complemented by a long-standing practice of project prioritization (amongst alternatives), patent protection, intellectual property audits, and enforcing patent rights aggressively. Although the company recognized employees in R&D for their creative output in terms of performance, the ideas developed were considered the intellectual property of Alpha-D; some were patented while others were kept in the company repository.

The acquired Brownfield employees became grouped together focusing on a single product line within a broader business group encompassing several products. The Lab operated as an independent entity, positioned as a specialist Lab and also a global resource to serve customers around the world. Along purely technical lines, the Lab sits as one of several R&D labs within the structure of Alpha-D. Archival records indicate that historically, labs were established in the company to be the engine of creativity and growth.

The Lab post-acquisition retained its historical subject area organization into software, hardware and support functions. More than two-thirds of the Lab team structure was involved in software engineering. Others were hardware engineers and quality assurance / testing technicians. Software engineering work was historically a dominant part of the team's work as it defined the value inherent in the product in terms of what was being offered to the customers.

## Findings and discussion: The vulnerability of practice-like activities

Whereas much MacIntyrean research within business ethics and organization studies has emphasized the “practice” element in practice-like work, we focus on what makes the addition of “-like” necessary, what that suffix tells us, and what its consequences are. Thus, what we offer is complementary to existing accounts of practices, but focuses on the negative side, the vulnerability of the “practice-like” activities that are a relatively widely available feature of working life. We do so by exploring a variety of themes that emerge from our study of the Lab. These include the goods of the Lab's work, particularly as they relate to the discussion of practices and practice-like activities above, the indifference of management to internal goods and the inconsistent frames of reference characterizing seeing things so differently between the Lab's practice-like positionality, emphasizing community, and the communal mastery of their craft. We also note the institution's emphasis on external goods, its indifference to practices and internal goods, and its push toward individual forms of performance management, aiming at external goods. Finally, we examine the ethically salient changes to the workplace community as a result of the takeover.

### Goods of the lab's work

Prior to the takeover, there were clearly pockets of work within the Lab that were highly practice-like as was evidenced by the researcher's study of historical aspects of the Lab and the acquired organization prior to their takeover by Alpha-D—from interviews with members of the Lab who were working together pre-acquisition. The following passage from Julian—part of the design team—demonstrates a degree of devotion and commitment we would expect from those seriously engaged in a practice:

I was working from home and so I had no distractions…yeah…I practically worked all day every day and I had, you know...just great...fantastic…to actually have that opportunity to just really focus, really get your head down and just design, design, design, design, design for like six weeks solid, and that design is still sort of the basis of the product today although it was 7 or 8 years ago probably. (Julian)

While long working hours is not necessarily evidence of practice-engagement, the evident joy taken in design here separates this from a competitive desire to show commitment in the manner of presenteeism. Indeed, Julian also articulated the goods of software design with analogies to architecture, which is a paradigmatic practice and one of the examples offered in MacIntyre's initial formulation of the concept.

I never know what a good analogy for software is...sometimes I talk about a building, but a big complicated building, not a simple little one, and you want to add another story on or you want to dig a basement or you want to add a wing or something, you know...you can do all of this… and if the original building was good, it has very strong pillars, and very strong foundations, then maybe it's easier to adapt it. If the original house was badly built, you add another story and the whole thing collapses. It's an analogy but it's not good I think, because in some way software sections are more complicated than a building. More like a machine with lots of interacting moving parts. (Julian)

Here we see a commitment to the ideal of craftsmanship emphasized by Moore (2005a). However, somewhat dissatisfied with the architecture analogy, Julian went on to compare software design to writing a book.

Software is extremely complex. You know you can end up with hundreds of thousands of miles of codes. So many infinite ways of...the languages are as expressive as human languages… somebody who writes a book, and somebody else writes a book, nobody is ever going to write the same thing. So, there is infinite creativity that software engineers can...which is both wonderful but also kind of scary. (Julian)

However, Julian's enthusiasm notwithstanding, there are certain characteristics of the work conducted at the Lab that suggest a status of “practice-like” is more appropriate than that of fully fledged practice.

Firstly, there is the fact that we are dealing with “practical and productive activities” (Hager, 2011, p. 548) which are often harder to make sense of as fully-fledged practices because of the commercial pressures they face. Put simply, they are hard to divorce from external goods, in the way that, for instance, painting, physics, and philosophy are not.

But there are further reasons in this case. Let us consider the characteristics of practices in turn. Practices are defined by MacIntyre as above, as being coherent, complex, socially established, cooperative activities, through which internal goods are realized “with the result that human powers to achieve excellence, and human conceptions of the ends and goods involved, are systematically extended” (MacIntyre, 2007, p. 187).

Clearly the software design undertaken in the Lab was coherent and complex. Software design is also socially established, to at least some degree. However, it is worth noting a distinction between the strong degree of social establishment which paradigmatic practices such as philosophy, physics, and poetry enjoy, and the somewhat reduced degree of social establishment of the kind of software design which took place in the Lab. Early experimenters in chronophotography—a Victorian era precursor of motion pictures—were not yet engaged in the practice of cinema because cinema was not yet an historically established practice. Consider Geertz's well-known definition of culture as “a historically transmitted pattern of meaning embodied in symbols, a system of inherited conceptions expressed in symbolic forms by means of which men communicate, perpetrate and develop their knowledge about and attitudes toward life” (Geertz, 1973, p. 89). Full social establishment would seem to imply something of this kind, and the work conducted in the Lab seems to be only partially in accordance with such a requirement.

Is software design cooperative? Notwithstanding Julian's enjoyment of at times working alone, software design is still a team effort for the most part. However, the competitiveness of the market hinders this to some degree. The agonistic orientation rival firms have to one another ensures the cooperative aspect is severely limited, even if it is present.

However, when we get to the “systematically extended” clauses, that is to say, the requirement that engagement in a practice results in a systematic extension of both “human powers to achieve excellence, and human conceptions of the ends and goods involved” (MacIntyre, 2007, p. 187), the status of the Lab's work seems to fall short of what we would expect from a clear and unambiguous example of a practice. This is largely a result of factors operating at the level of the institution and the level of the broader economic realities of society.

The requirements that human powers to achieve excellence and our conceptions of the ends and goods involved in the practice are in tension with restrictions imposed by intellectual property especially since, as data analysis revealed, there was a conscious effort by management for turning the creative elements of personal and teamwork into patented intellectual property (linked to a pursuit of external goods at the level of practice-like activities). Clearly, not all products of practices are openly available to all—one might need money to purchase works of literature or pay for access to online academic articles. However, in these cases there are public libraries, and common practices of sharing and disseminating research, that make the products of practices more widely available. Certainly, the restrictions placed by intellectual property on software design are an order of magnitude more severe than in such cases. This is why open-source software is so appealing to software practitioners (see Von Krogh et al., 2012). Intellectual property restrictions have the effect of transforming the internal goods into quasi “external goods,” goods which are “always some individual's property and possession” (MacIntyre, 2007, p. 190).

As MacIntyre notes, “when Turner transformed the seascape in painting or W.G. Grace advanced the art of batting in cricket in a quite new way their achievement enriched the whole relevant community” (MacIntyre, 2007, p. 191). Such extensions were impossible for the Lab, because the rest of the community of software designers, i.e., those outside the organization, are deliberately excluded from understanding the goods in question. This “practice-like” status renders the goods of the Lab's work particularly vulnerable to being eroded by changes to the community instigated by management after the takeover.

So, as the data reported suggests, the activity possesses its own distinctive internal goods. However, these goods are in particularly acute danger of being unnoticed by the outside observer. This is a recurrent problem with productive activities and reminds us that classification of practices is not merely an a priori exercise. Precisely how an activity is engaged with, and how it is available for engagement, shapes its status. This is evident in the “disquieting suggestion” with which *After Virtue* opens. Here, MacIntyre describes a scenario in which the natural sciences have suffered a catastrophe which leads to scientific knowledge being largely lost, and scientific debates are conducted in a way that they are not “natural science in any proper sense at all” (MacIntyre, 2007, p. 1). MacIntyre uses this vivid example as a metaphor for ethics in modernity, and yet it clearly illustrates the vulnerability of practices to contextual factors too. The natural sciences are clearly practices, and yet as MacIntyre's example suggests, someone can be engaged in an activity that they take to be a practice, that appears to an observer to have many important features definitive of that practice, and yet that practice, properly understood, may remain unavailable to them.

### Indifference of management to internal goods, its positionality and frames of reference emphasizing mastery of external goods at micro and institutional levels

The critique of management is a long-standing theme in organization studies (see, for instance, Braverman, 1974; Anthony, 1977), with MacIntyre's critique of management as manipulative, which we outlined above, being an important current. However, as we saw, Moore plausibly rejects this as a picture of management *per se*. Nevertheless, the point and purpose of management, which is, in part at least, to secure external goods, makes a focus on internal goods difficult to achieve. The pressures and incentives managers face encourage them to prioritize institutional goods even at significant cost to goods of practice, including the sustaining of community and craft which were more salient in the Lab. This was the case in the Lab, where institutionally imposed deadlines and test requirements—box ticking, more speed, less quality—were often in tension with the goods of the core practice (or practice-like activity), even where these goods and the interests of the customer align:

The release manager who's really difficult, a difficult man who's always very frustrated ‘cause we are always missing dates. Yeah, it was really stressful. (Alex)

You need a test plan, and you need to execute that test plan and we need to see the results and the results must be stored in the repository. Somebody's saying you can go back and refer to that. What it doesn't say is what is in the test plan. So, your test plan can literally say... ‘Switch it on. Can you see images coming through? Yes.' You've passed the test…You can always put anything in the test plan because the overriding quality procedures...or whatever, processes that we have to conform with are saying you need a test plan, you need to execute it, it needs to pass and you need to store results in the repository and it's good to have all those things. But there's a missing bit that that process can't do and that's the person who's writing that test plan. Is he writing the test plan so that the thing will pass? Or is he writing the test plan to genuinely challenge the product, so that we are that much more confident of the quality? The problem with writing test plans that are too challenging is that you may be uncovering problems that you'd rather not know about. But I still say it's better to uncover them than to have it out in the field when the customer uncovers the problem. (Julian)

However, while MacIntyre's critique of management may prompt us to look for ethical failure in management, the situation after Alpha-D took over the Lab may be understood as a primarily epistemic problem. After all, practices are properly appraised only by those with significant first-hand knowledge (MacIntyre, 2007, p. 189). However, with more established or paradigmatic practices, the internal goods are visible to the outside observer to at least some degree. A cursory understanding of unfamiliar sports and art forms, for example, are often enough for us to make confident judgements about the status of such activities as practices, whereas this is not so with more marginal cases. Practitioners of relatively “nearby” practices, i.e., the field-hockey player is likely to have a working grasp of the goods of ice-hockey (Sinnicks, 2019). Likewise, those who institutionally safeguard clear examples of fully-fledged practices, i.e., the manager of a firm of architects will understand the goods of architecture (Moore, 2017). In the case of the Lab, however, the Alpha-D management seemed to be largely unaware of the goods of the activity in question, and at times entirely indifferent to it, which further supports our categorization of the work undertaken at the Lab as practice-like, rather than an example of a fully-fledged practice.

In the following passage, Alex, who worked in quality assurance, is reflecting on why a Lab sub-unit lead and his team seemed so hostile:

so why are they so angry with me, I don't know, and to be fair that project [involving a legacy Brownfield product that one of the Lab team leads was working on] is a very low priority, there hasn't been any real, for a long time we were going to just…um stop it… they wanted to finish it off properly, like test it and see whether it performs well and then kind of shelve it for the time being. So, of all the compliance regulatory stop sale issues I have going on, that thing isn't even on my radar. You don't ask me to schedule a test for something that we want to shelve, when I know that everything is in place for them to get what they need.

For a long time Julian was quite fond of it because he thought that we should be doing it and it's all about not just reading number plates but reading hazard plates like erm… for lorries if it's carrying flammables or explosives…that kind of stuff…it's reading those side triangles… Marketing in the past had asked, ‘why in God's name are you working on that, nobody has even asked for that right now'… (Alex)

In the classic case of institutional domination, the activity housed in the institution is a practice but is being poorly sustained by that institution which has drifted toward a preoccupation with external goods. Think of the football club which comes to disproportionately value the next sponsorship deal, the next summer friendlies tour of a location with a growing market, rather than the goods of football. Such a case illustrates what happens when institutions dominate practices. The case of the Lab is quite different. In this case, the goods of the activity barely even register, they are not visible to Alpha-D in the way that the goods of football remain visible even to the most institutionally dominated football club, which again suggests the categorization of the work as “practice-like” is apt.

Besides the indifference of management to internal goods we may observe inconsistent frames of reference between the institution and the Lab. As suggested by the work of Bolade-Ogunfodun et al. (2022) on nuanced and interacting factors which inform interpretive standpoints, the positionality of the Lab (practice-like) informs its understanding of its role and activities. On the other hand, the institution's positionality frames how it sees its relationship with the Lab; a relationship which emphasizes external goods, coupled with what appears to be almost indifference to practices and internal goods. The institution accordingly chose management and expected their roles to function in a way which materializes the intentional effort to replace communal loyalty into individualized forms of performance and accountability. In addition, the Lab management were expected to work in alignment with the institution's emphasis on external goods, reflected in the acquisition of intellectual property *via* transformation of creative outputs of work teams into company intellectual property; all of which created ethically salient changes to the workplace community as a result of the takeover.

That the new management appears to be largely unaware of the nature of the Lab's project is telling. The extension of goods, and conceptions of goods, that Julian's work could, under the right circumstances, contribute to is not fully recognized by the institution, as Alex's comment above makes clear. Where practice-sustaining institutions come to allow institutional goals to dominate, there is often lip-service to the goals of the practice, but in this case, they are outside the scope of the institution's aims and objectives, and indeed, do not even register as a concern.

### Changes to the workplace community

The Lab's overall business workforce consisting of engineering staff (i.e. the Lab), customer service and technical support teams were located on the first floor of the company's large office complex in the UK. Recollections of participants during interviews revealed that prior to and in Brownfield, the engineering team worked within the same small office and had informal social relations. Non-engineering staff sometimes took on supportive roles outside of their formal roles to speed up the process of packaging and shipping the products to customers. In Alpha-D however, core engineering staff in the Lab sat on one side of the floor separated from their other former Brownfield colleagues by a thick partitioning. There were rumors amongst legacy Alpha-D employees in the division that the Lab had specifically requested to be positioned away from the generality of Alpha-D colleagues who sat in the company's usual open plan arrangement. On the other side of the partitioning, technical support and customer service teams sat around desk hubs along a row of hubs occupied by other Alpha-D employees (business and sales, other product groups).

All practices are vulnerable to institutional acquisitiveness, but activities which are merely “practice-like” are especially so, particularly in light of the importance of the community-focused aspect, as discussed above. The way the post-acquisition changes altered the sense of community is a clear theme in the data.

Months (emphasized)… it took them months to get connectivity, to get permission to go out there on the pole… they had to have safety training… So [Alpha-D] as a company has, you know, health and safety rules… This team was not used to dealing with traditional company bureaucratic journals... you had to fill out this form and you follow up and then you go say, ‘hello can I put in this form for this? Can I get this done?'…you know they don't work that way. They were a small company shop where if they wanted something, they just do it, you know (Alex)

Here we see evidence of a more informal culture that the workplace community enjoyed prior to the takeover, and how the practice-like activity was sustained by it. Such an informal culture is not something that the new management, after the takeover, could properly understand. Practices are only understood by the initiated, but the same is also true of communities which sustain both practices and practice-like activities. The new management overseeing the Lab inevitably lacked the necessary contextual understanding available only to participants within that community.

As we noted above, the particularities of the community are especially important in cases of practice-like activity, where it seems that the practice-like activity could be substituted without loss provided the community remains stable. The community connected to the Lab was undermined by the changes resulting from the takeover. As other participants put it, when talking about how things worked pre-acquisition:

When we were in Brownfield… you had your role… and one way or another we turned round our equipment very quickly. We had quite good customer services… but with this company obviously you are part of a massive company, it's more procedures, more processes. You can't do anything that isn't your role and it's frustrating sometimes. (Fran, Customer services team member)

Just the size of the team itself made it seem very family. (Jody, Lab sub-unit member).

Here we see the growth in scale as a problem, which is unsurprising if MacIntyre's (1999) emphasis on small communities is warranted. The changes to the community saw a decline in the feeling of closeness that had previously existed prior to the takeover. Again, this is a particular problem for communities attached to practice-like forms of work which, *ex hypothesi*, lack the especially noteworthy internal goods of more paradigmatic practices, and which might allow the community to cope with a significant expansion in size, in the way that a growing chess club or community choir might. In the absence of such goods, peer relations are particularly vital to the sustaining of the goods of community, and indeed, given the absence of such goods in this case, the community of the Lab was clearly undermined by the growing scale of operations after the takeover:

Yeah and obviously we've been sort of a close knit… and it wasn't perfect but you get used to working closely with this people and all of a sudden they say the Lab are a global resource so we can't sort of really go and talk to them, like we would just go upstairs and say “oh we need this” or “can you advise me on that” or… and now you are aware that obviously they are doing things for all over the world, like everybody is all different departments, different people, but it's suddenly weird to be segregated (Fran, Customer services team member).

This passage also reflects a decline in levels of participation, an important MacIntyrean good as we noted above, in the wider work.

## Conclusions

In this study we have utilized empirical evidence to illustrate how the concept of “practice-like” activities differs from paradigmatic practices suggesting their increased vulnerability to institutional domination. We also showed how the practice-like nature of the studied work activities and their vulnerability are exacerbated by institutions which operate under a strong capitalist logic. We offer further support to MacIntyre's critique of management in light of how, in the aftermath of the acquisition, institutional concerns took attention away from the goods of the acquired engineering company, and its practice-like Lab.

Given its suitability to detailed and nuanced examinations of particular forms of work, ethnography has proven to be a particularly apt methodology for conducting empirical research in this study and, more widely, as part of a MacIntyrean framework. Indeed, a number of existing ethnographies that do not adopt an explicitly MacIntyrean approach can nevertheless be fruitfully read in MacIntyrean terms. See, for instance, Wacquant (2022), a work which makes no reference to MacIntyre, and yet which nevertheless brings the internal goods of boxing vividly to life. Likewise, McCann's (2022) study of the paramedic profession. Such ethnographies are not written with MacIntyre's “virtues, goods, practices, and institutions” (Beadle and Moore, 2006) schema in mind, and yet provide accounts of activities that lend themselves to such an analysis.

Given MacIntyre's own work which expressly addresses business (MacIntyre, 1982), management (2007, ch. 3), and practices (2007, ch. 14), as well as the seminal contributions made by the likes of Moore and Beadle, it is perhaps inevitable that MacIntyrean research in the field has tended to focus on business, management, and the goods of various occupations. There have also been papers that focus on topics such as leadership (e.g., Kempster et al., 2011; Mensch and Barge, 2018; Sinnicks, 2018), as well as some impact on the organizational learning literature (Halliday and Johnsson, 2010); nevertheless, there is clearly scope for MacIntyre's work to be fruitfully brought to bear on other topics in organization studies (though see Beadle and Moore, 2011, for an important contribution in this vein), in particular, organizational culture. Drawing on Schein (1983, 1984) as well as a variety of other commentators on corporate culture, Moore (2005b) insightfully argues that “culture and values” represent the institutional equivalents of “character and virtues,” which are available through practice-engagement. As a result, Moore takes a greater interest in the latter pair of concepts. However, in line with our recommendation that researchers pay more attention to management outside of practices, we also recommend that closer attention is paid to culture and values, which are likely to need more deliberate attention when the organization is not structured around sustaining a clear practice.

Part of the argument developed in the present paper is that paradigmatic practices have such an obvious appeal that, other things being equal, they tend to facilitate a culture in which their goods are central to the organization. While institutional pressures can be very real, and can take attention away from the core practice, they can be resisted. While football clubs care a great deal about merchandise sales, sponsorship rights, and so on, they usually do also care a great deal about football. While arts venues are not shy about trying to maximize revenues from sales of over-priced refreshments, and sometimes cannot resist the temptation to over-programme bland but popular repertoire, they nevertheless usually clearly care about the arts. The same clear emphasis on internal goods is not available to “practice-like” activities. Indeed, while those engaged in paradigmatic practices might reasonably be expected to care a great deal about them, this is not true of people whose work requires them to carry out a practice-like activity. Thus, while we might expect a broadly similar set of motivations and preoccupations amongst, for instance, footballers and orchestral musicians, the same is not the case for employees who work in exactly the same field where that field is only practice-like.

As empirical data in this case suggest, in the case of practice-like work, the practice-like element is partially dependent on the peer relationships, and self-understanding that accompanies, or is made available, by the activity. One and the same activity may be more or less practice-like depending on how it contributes to this communal aspect, which is one reason why “workplace change” is liable to be ethically troubling: it can disrupt the relationships that foster the communal aspect. Hence, organizational culture becomes a particularly important topic, as well as friendship and the achievement of a common good.

Relationships within the workplace often contain aspects of Aristotle's (2000) three types of friendship: virtue, pleasure, and utility, and indeed can provide an indication of how the three might come together. We find our colleagues useful in helping us to complete our daily tasks, we take pleasure in working with them, and we value the virtues of those who contribute to the shared endeavor. As MacIntyre notes, approvingly citing Aristotle and Aquinas, “trust is among the requirements for friendship” (MacIntyre, 2010, p. 10), as is fidelity (2007, p. 123). Both the trust required for friendship, the long-term relationships in which fidelity can be expressed, and friendship itself, are vulnerable to being undermined by the kinds of workplace changes described above.

Since human flourishing requires virtuous friendship (Aquinas, 2006, S.Th. I-II q.4, a.8; II-II q.47 a.10, ad.2), some kind of cooperation in terms of fellowship is necessary to overcome an individualistic, self-interested approach to human work, which leads to an ethics of effectiveness and personal advantage (Horvath, 1995). As MacIntyre puts it, when commenting on Adam Smith, “[w]hat is missing from Smith's account is any conception of economic activity as capable of being cooperatively and intentionally directed toward the achievement of common goods” (2016, p. 92). In the workplace, this form of friendship can be considered as “fellowship.” As Pinto et al. state following MacIntyre (1999, p. 150), “fellowship (while not literally in MacIntyre's vocabulary) can help us understand how the concept of the common good can be introduced into modern corporations as a narrative of benevolence (friendship) based on shared activity in the workplace” (Pinto-Garay et al., 2022, p. 259). Hence, a narrative of fellowship with colleagues is a form of participating in a common good, in other words, a way to make practice-like activities in the workplace common good-oriented.

According to MacIntyre, “the common goods of those at work together are achieved in producing goods and services that contribute to the life of the community and in becoming excellent at producing them” (MacIntyre, 2016, p. 170). Contributing to the common good by doing one's worthwhile job well, even if that job is not a practice, or even practice-like is valuable, and is something that can be aided by good management. In the society that desperately needs turnips, the turnip-planter is making a valuable contribution even if he or she is not directly engaged in a practice while at work, and yet the turnip-planter's job can be made better or worse by good or bad management (see Sison and Fontrodona, 2012; Sison, 2016). Thus, and finally, it is worth reflecting on what might constitute good management outside of practice-based work, taking into account that every common good can only be achieved collaboratively and that both the goal and the path of ethics as practical reasoning are common goods (MacIntyre, 2016).

## Data availability statement

Research ethics and data anonymity and confidentiality protocols do not allow access to the first level of ethnographic data as they may contain information which could run the risk of identifying the participants. The raw data supporting the conclusions of this article in their fully anonymised version can be made available by the authors, without undue reservation. Please contact YB-O for access to these data.

## Ethics statement

The studies involving human participants were conducted following formal ethics approval at the academic institution of Author 1. Ethics protocols were reviewed and approved by Henley Business School, the University of Reading, via Professor Evelyn Fenton, who at the time of the study in 2015–2016 was the School Research Director. No ethical concerns were raised nor were missing information indicated, so the ethical approval was given at School level (first instance). Consent was also obtained from the participating organization and individual research participants, who were all fully anonymised so that no one is identifiable. The participants provided their written informed consent to participate in this study. Following data analysis all participant information which could potentially identify them was removed and data fully anonymized. Author 1 to who the data belongs maintains record of this data and appropriate data management processes.

## Author contributions

YB-O has made the data available from the ethnographic study. All authors contributed to the article and approved the submitted version.
